# An abuse liability assessment of the glo tobacco heating product in comparison to combustible cigarettes and nicotine replacement therapy

**DOI:** 10.1038/s41598-022-19167-8

**Published:** 2022-08-29

**Authors:** George Hardie, Nathan Gale, Michael McEwan, Stefano Milleri Oscar, Luigi Ziviani, Christopher J. Proctor, James Murphy

**Affiliations:** 1 British American Tobacco (Investments) Limited, Research and Development, Regents Park Road, Southampton, SO15 8TL UK; 2 Centro Ricerche Cliniche di Verona, Policlinico G. B. Rossi, P.Le L. A. Scuro 10, 37134 Verona, Italy; 3 DoctorProctorScience Limited, 157 Cavendish Meads, Sunninghill, Ascot, SL5 9TG UK; 4 R. J. Reynolds Tobacco Company, 401 N Main Street, Winston Salem, NC 27101 USA

**Keywords:** Biomarkers, Medical research

## Abstract

Tobacco heating products (THPs) have reduced emissions of toxicants compared with cigarette smoke, and as they expose user to lower levels than smoking, have for a role to play in tobacco harm reduction. One key concern of Public Health is that new tobacco and nicotine products should not be more addictive than cigarettes. To assess their abuse liability, we determined nicotine pharmacokinetics and subjective effects of two THPs compared with conventional cigarettes and a nicotine replacement therapy (Nicotine inhaler). In a randomised, controlled, open-label, crossover study healthy adult smokers used a different study product in a 5 min ad libitum use session in each of four study periods. Product liking, overall intent to use again, urge for product and urge to smoke questionnaires were utilised to assess subjective effects. Nicotine uptake was greater for the cigarette (C_max_ = 22.7 ng/mL) than for either THP (8.6 and 10.5 ng/mL) and the NRT (2.3 ng/mL). Median T_max_ was significantly longer for the NRT (15.03 min) than for the tobacco products (4.05–6.03 min). Product liking and overall intent to use again was highest for the cigarette, and higher for the THPs than the NRT. Urge to smoke was reduced more by the cigarette than by the other three products. Urge to use the THPs was greater than the NRT. These findings suggest that the abuse liability of the THPs lies between that of subjects usual brand cigarettes and the NRT.

## Introduction

### Background and objectives

Nicotine, a chemical found naturally in tobacco leaf that is transferred to cigarette smoke during combustion, is primarily responsible for the addictive properties of cigarette smoking^1^. However, nicotine is not considered to contribute substantially to smoking-related diseases^2,3^, which generally result from the inhalation of tobacco smoke containing thousands of chemicals^4^ and numerous toxicants^5,6^. When a smoker inhales cigarette smoke, nicotine is rapidly transferred to the bloodstream and transported around the body; in the brain, it activates neuronal nicotinic receptors involved in mood and relaxation, which along with the sensorial aspects of smoking, results in the pleasurable and rewarding effects experienced by a smoker^7^.

Pharmaceutical nicotine products such as nicotine replacement therapies (NRTs) aim to replace the nicotine supplied by conventional cigarettes and thereby assist individuals in stopping smoking by reducing cravings, symptoms of withdrawal, and mood changes^8–10^. In general, however, the delivery of nicotine from NRT products is relatively slow and the pharmacokinetic (PK) profile does not resemble that of cigarettes^11–14^. The time to maximum plasma nicotine concentration (T_max_) tends to be longer, while the maximum nicotine concentration (C_max_) is not characterised by the sharp peak seen with cigarettes, but by a lower and flatter peak^12–14^. As a result, smokers do not achieve the same nicotine levels or satisfaction with NRT products that they do when smoking cigarettes. NRT is also sensorially deficient as compared to cigarette smoking in terms of puffing ritual and cues associated with smoking, and as a result of the PK profile, it has a lower abuse liability compared to cigarette^15^.

A recent review of more than 100 trials concluded that NRTs can increase the rate of successful quit attempts by 50–60% for smokers who want to quit^16^, but they do not do so for all smokers, potentially due to the slower and reduced delivery of nicotine relative to cigarettes^8,9,17^, the sensorial deficiencies of NRT and because they do not replace the behavioural activities of smoking [16. Notably, NRTs are considered medicinal products whereas tobacco heating products and other non-combustible products are still considered consumer products and are not approved for smoking cessation. It is therefore important to complement existing cessation initiatives with strategies that attempt to reduce or prevent harm in those who would otherwise continue to smoke.

Tobacco harm reduction, where smokers who are unwilling or unable to quit smoking cigarettes replace cigarette smoking with the use of nicotine and tobacco products with potentially fewer health risks^18,19^, is a strategy that—if widely adopted—might potentially offer substantial public health gains through avoidance of projected tobacco-related harm^19^. For many years, tobacco researchers and policy experts have embraced the idea that alternative sources of nicotine that provide rewarding effects similar to those of cigarettes might be used to encourage smokers to switch away from cigarette smoking^19^. In this regard, tobacco heating products (THPs) are electronic devices that heat, rather than combust, tobacco contained in a consumable “stick”, producing an aerosol that the user inhales^20^. Owing to the lack of combustion, many of the toxicants found in cigarette smoke are absent or present at significantly reduced levels in the aerosol generated by THPs^21,22^. Preclinical evidence further indicates that the THP aerosol has reduced in vitro biological activity compared with cigarette smoke^21,23–25^, while several clinical studies have shown significantly reduced toxicant exposure and favourable changes in biomarkers among adults who switch from smoking to using THPs^26–30^.

Although information on the chemical emissions from THPs and preliminary toxicological data are emerging, relatively little is known about consumer behaviour and/or the abuse liability (dependence potential) of these products, i.e., the likelihood of engaging in persistent and problematic use of alternative nicotine products resulting in undesirable consequences including physical and/or psychological dependence^31,32^. This information is especially important because any product that fails to deliver nicotine satisfactorily, or conversely demonstrates an abuse liability profile such that non-users may be more likely to initiate and develop sustained product use, may significantly undermine any potential for harm reduction. As a result, the US Food and Drug Administration (FDA) requires an abuse liability assessment, including exposure to nicotine during use and evaluations of misuse potential, as a component of Premarket Tobacco Product Applications for new tobacco products^33^.

The abuse liability of a tobacco or nicotine product can be determined both by assessing the speed and quantity of nicotine delivery, with higher abuse liability observed for products providing greater delivery, faster absorption and higher plasma concentrations of nicotine, and by assessing subjective effects such as appeal, responses to products and product acceptability^31,34–36^. Subsequently, an approach based on such methods, including an assessment of nicotine PK and subjective effects, has been used to determine the abuse liability of several e-cigarettes (e.g.,^13,14,37–40^). However, while nicotine PK data for THPs has previously been reported^41,42^, there is a paucity of literature concerning the abuse liability of these products coming from studies in which it has been rigorously assessed. In this study, therefore, nicotine PK and various subjective effects indices (product liking, intent to use again, urge to smoke, urge to use the THP, satisfaction, and evaluation of other both positive and negative effects) have been evaluated to determine the abuse liability profile of the glo THP in relation to two products on the extremes of the tobacco and nicotine-containing product risk continuum^43–46^, namely combustible cigarettes, which have high abuse liability, and NRT, which has low abuse liability^34,45,47^.

## Methods

### Study design

This was an open-label, randomised, crossover, four-treatment, four-period, single-dose clinical study in which healthy adult smokers were recruited to receive one of four investigational products (IPs) during each of four study periods. Blood samples were taken for nicotine PK analysis and participants completed various subjective effects questionnaires. The study was conducted at the Centro Ricerche Cliniche di Verona (Verona, Italy) in July to September 2018. Study authorisation was received from the Italian Medicines Agency (AIFA) based on review by the Italian National Health Institute (reference 1630) and was also approved by the Ethics Committee for Clinical Trials of the Provinces of Verona and Rovigo (reference 1778CESC). The study was conducted in compliance with the ethical principles of the Declaration of Helsinki, Good Clinical Practice (International Conference on Harmonisation E6 Consolidated Guidance, April 1996) and Italian laws, including those relating to the protection of personal data. All participants provided written informed consent prior to any study procedures. The study was registered in the ISRCTN and EudraCT databases (references ISRCTN13439529 (07/08/2018) and 2018-000701-23, respectively).

### Participants

The participants were healthy adult smokers (aged 19–60 inclusive) of at least 10 non-menthol, combustible cigarettes per day who had been smoking for at least 1 year and had been smoking their usual brand of cigarette for at least 6 months. Potential participants attended a screening session, where smoking status was confirmed by exhaled carbon monoxide (eCO: ≥ 10 ppm) and urinary cotinine (≥ 200 ng/ml) assessments.

The main exclusion criteria were pregnancy or breastfeeding; non-agreement to using contraception for the duration of the study (female participants); acute illness (e.g., upper respiratory tract or viral infection) requiring treatment within past 4 weeks; use of nicotine or tobacco products other than commercially manufactured cigarettes within the past 14 days; self-reported non-inhalation of cigarette smoke; medical history of asthma or chronic obstructive pulmonary disease (COPD); use of bronchodilator medication within the past 12 months; blood donation of 450 ml or more within past 90 days; and clinically relevant abnormal findings on physical examination, medical history, electrocardiography, lung function tests, or clinical laboratory panel. Individuals were also excluded if they were planning to quit smoking in the next 12 months. All participants were free to quit smoking and withdraw from the study at any time. Any individual who decided to quit smoking was directed to appropriate stop smoking services.

### Study products

The THP (glo, British American Tobacco) consists of two components: an electronic heating device, and a tobacco consumable rod (neo stick). Two variants of the Neo THP consumable rod with different nicotine contents were used with an identical heating device: The THP1.0(RT) consumable rod yielded 0.46 mg nicotine/stick and THP1.1(RT) yielded 0.68 mg nicotine/stick, as measured under a modified Health Canada Intense machine smoking regime (55 mL puff volume, 30 s puff interval, 2 s puff duration, 8 puffs, no vent blocking)^22,48^. Both variants of the THP consumable rod were tobacco flavoured but differed in blend type (1.0 and 1.1).

The first reference product was a NRT (Nicorette Inhalator; 15 mg nicotine; Johnson & Johnson). Nicorette delivers nicotine via the inhaled route and attempts to replicate some of the sensorimotor cues associated with smoking. The second reference product was each participant’s usual brand of combustible cigarette, as reported at screening and supplied by the participants themselves. The THP devices and consumables and NRT were provided free of charge to participants.

### Randomisation procedure

Randomisation sequences were prepared by Cromsource Srl (Verona, Italy) with SAS statistical software version 9.3 (SAS Institute Inc., Cary, NC) and using a Williams latin square design composed of 8 blocks of 4 subjects. In ascending order of subject number, enrolled participants were assigned to receive the four study products in accordance with the pre-defined randomisation sequences, with an equal proportion of participants in each sequence.

### Study procedures

Individuals who fulfilled the inclusion/exclusion criteria at screening (visit 1) were enrolled in the study and basic demographic characteristics and information on cigarette consumption were recorded. Participants then attended a randomisation visit (visit 2), where the sequence of study product use was assigned as described above. For approximately 1 week before their PK session for THP1.0(RT), THP1.1(RT) or the NRT, each participant was provided with the relevant study product (glo THP device and one pack of 20 of the relevant neo sticks, or one box of NRT inhalator and consumables) and asked to familiarise themselves with its use before attending the session.

Participants were admitted to the clinic on the evening before each PK session and their eligibility was reconfirmed. They abstained from nicotine or tobacco product use overnight (at least 12 h). On the following morning, a baseline blood sample was taken (–5 min) by direct venepuncture and then participants used the assigned study product for 5 min. Puffs were counted during use and blood samples for plasma nicotine analysis were obtained at 1, 3, 4, 5, 6, 7, 9, 15, 45, 90, 180 and 240 min relative to the first puff on the study product. Plasma samples were analysed for nicotine by ABF GmbH (Planegg, Germany) using an Acquity UPLC equipped with a Xevo TQ-S triple quadrupole mass spectrometer. The lower limit of quantification for nicotine using this validated method was 0.1 ng/mL. Heart rate, blood pressure, respiratory rate and body temperature were also measured throughout each PK assessment period.

At various points before, during and/or after the product use session, participants completed five questionnaires. The product liking questionnaire (PLQ) asked “At this moment, how much do you like the product?” (ranging from 0 [strongly dislike] to 5 [neither like nor dislike] to 10 [strongly like]) and was administered at -5, 3, 5, 9, 30, 60, 120 and 240 min. The overall intent to use again (OIUA) questionnaire was administered at a single timepoint (240 min) and asked participants to “Rate the degree to which you would like to use the product again” (ranging from 0 [not at all] to 10 [very much]). The urge to smoke (UTS) questionnaire asked “How strong is your current urge to smoke your usual brand cigarette?” (ranging from 0 [no urge] to 10 [extremely strong urge]) and was administered at -5, 3, 5, 9, 15, 30, 45, 60, 90, 180 and 240 min. The urge for product (UFP) questionnaire was completed only after use of the THPs and NRT (at -5, 15 and 120 min) and asked “How strong is your current urge to use Investigation Product?” (ranging from 0 [no urge] to 10 [extremely strong urge]). The product evaluation scale (PES)^48^ was also used to assess subjective responses to the study products and was administered at -5, 15 and 240 min.

**Figure 1:**
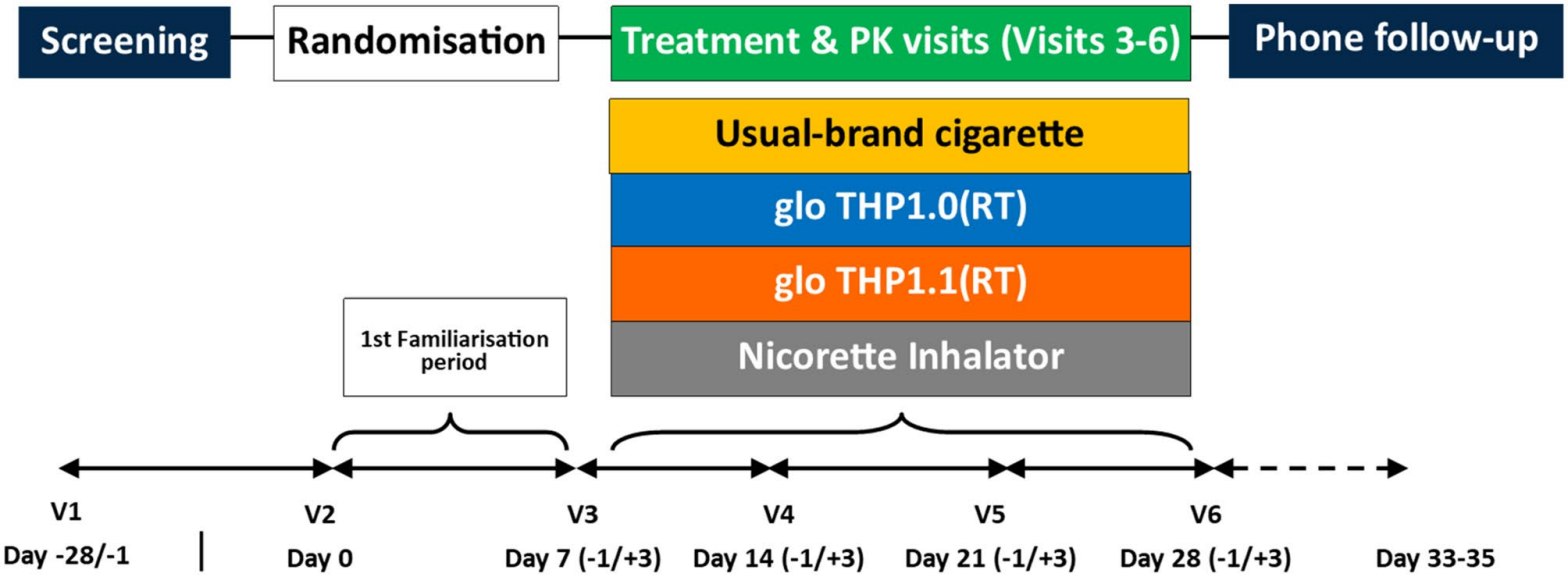
Schematic diagram of the study design.

Participants were discharged from the clinic after the nicotine PK assessment was completed. No later than 1 week after the final clinic visit, they were followed up by telephone to capture any post-study adverse events (AEs). (Fig. 1).

### Study endpoints

The primary endpoints were plasma nicotine PK parameters (C_max_, T_max_ and AUC_0-240 min_), PLQ score, OIUA score, UTS score and UFP score. Secondary endpoints were puff count during the 5-min ad libitum product use session and product evaluation as measured by individual item scores in the PES. Safety endpoints included adverse events and vital signs assessments.

### Sample size determination

The sample size was determined as 32 based on assumptions concerning all seven endpoints (AUC nicotine, C_max_, T_max_, OIUA, PLQ, UTS and UFP) at 90% power as outlined in Supplementary Table 1.

### Statistical methods

Statistical analyses and data processing were performed by using SAS software version 9.3. The PK analysis was performed by KinetAssist Ltd (Quothquan, United Kingdom) using Phoenix WinNonlin version 8.0 (Certara USA, Inc., Princeton, NJ).

AUC_0-240 min_ and C_max_ values were log-transformed and an analysis of variance (ANOVA) model with treatment, sequence and period as fixed effects and subject-within-sequence as a random effect was implemented. Ratios of estimated treatment population geometric means and their associated 90% confidence intervals (CI) were calculated from the exponential of the least-squares means difference and the corresponding 90% CI. Non-inferiority of the THPs versus the NRT was confirmed if the lower bound of the one-sided 90% CI for the ratio was at least 0.80. Non-inferiority of the THP versus the usual brand cigarette was confirmed if the upper bound of the one-sided 90% CI for the ratio was 1.25 or lower. For T_max_, Hodges-Lehmann nonparametric pairwise estimate of location shift between the THPs and NRT was performed. Faster nicotine absorption from the THPs compared with the NRT was confirmed if the upper bound of the one-sided 90% CI for the difference (THP – NRT) was less than -4.

PLQ, UTS, UFP and OIUA scores were log-transformed and an ANOVA model including treatment, sequence and period as fixed effects, and subject-within-sequence as a random effect, was implemented. The ratio of the adjusted geometric means between the THP and NRT was calculated with its one-sided 90% CI. For PLQ and UTS, the AUC of values from 3 to 240 min was used; for UFP, the mean of the values from 15 to 120 min was used; and for OIUA, the actual values at 240 min were used. PLQ and OIUA were tested for non-inferiority between the THP and the NRT, with non-inferiority of the THP confirmed if the lower bound of the one-sided 90% CI for the ratio (THP/NRT) was greater than 0.80. UTS was tested for superiority between the THP and Nicorette Inhalator, with superiority of the THP confirmed if the upper bound of the one-sided 90% CI for the ratio (THP/NRT) was less than 1.0. UFP was tested for superiority between the THP and NRT, with superiority of the THP confirmed if the upper bound of the one-sided 90% CI for the ratio (THP/NRT) was greater than 1.0.

The rationale for using a non-inferiority test is that we’re not interested in THP being significantly more liked than NRT, rather, we’re interested in THP not being less liked. We’re trying to show (at least) equivalence to the NRT (or better, which can be higher or lower depending on the parameter of interest). If we provide evidence for non-inferiority, this shows THP is accepted to the same level as NRTs and could thus provide a good alternative to NRTs in reducing cigarette smoking.

## Results

### Study population

The study enrolled 32 healthy male (n = 23) and female (n = 9) smokers aged between 22 and 57 years, all of whom completed the study and used all study products. Demographic characteristics of the study participants are summarized in Table 1. Among the 32 participants who enrolled, the mean ± SD age was 35 ± 8.9 years for men and 37 ± 11.9 years for women. Participants were current smokers of, on average, 17 cigarettes per day, with mean FTCD score of 6.0 ± 1.5.

**Table 1:** Demographic characteristics of study participants.

Characteristic	Men (n = 23)	Women (n = 9)	All participants
Age (years)	35 (8.9)	37 (11.9)	36 (9.7)
Weight (kg)	79.7 (9.3)	63.2 (8.3)	75.1 (11.7)
BMI (kg/m^2^)	25.9 (2.5)	23.7 (3.2)	25.3 (2.8)
FTCD score^a^	5 (1.6)	6 (1.3)	6 (1.5)
Cigarettes/day^b^	17 (6.8)	16 (3.5)	17 (6.0)

Values are presented as mean (standard deviation).

*BMI* body mass index.

^a^Fagerström test for cigarette dependence (FTCD) score at screening.

^b^Self-reported daily cigarette consumption at screening.

### Nicotine pharmacokinetics

**Figure 2:**
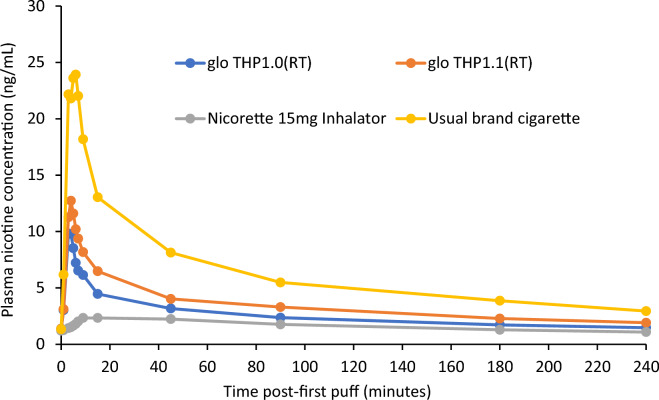
Mean plasma nicotine concentrations at each timepoint. Seven subjects were excluded from the PK analysis population due to major protocol deviations (washout problem), therefore n = 30 for usual brand cigarette, n = 31 for glo THP1.0(RT), n = 30 for glo THP1.1(RT) and n = 30 for Nicorette 15 mg Inhalator.

Regarding PK assessments, the mean plasma nicotine concentration at any time point was higher for subjects’ usual brand cigarette than for any other study product (Fig. 2). Plasma nicotine concentrations were also higher after use of THP1.0(RT) or THP1.1(RT) than after use of the inhalator NRT, while use of THP1.1(RT) resulted in higher plasma nicotine levels as compared with THP1.0(RT), which is consistent with the higher machine yield for nicotine of THP1.1(RT). Geometric mean C_max_ for the cigarette (22.7 ng/mL) was twofold higher than that for either of the THP variants (8.6 and 10.5 ng/mL) and tenfold higher than that for the NRT (2.3 ng/mL) (Table 2). Notably, median T_max_ was comparable for the cigarette (6.03 min) and the two THPs (4.05 and 4.07 min) but was much longer for the NRT (15.03 min).

**Table 2:** Plasma nicotine pharmacokinetic parameters measured among adult smokers following 5-min ad libitum use of the glo THP, usual brand cigarette or NRT.

Parameter	Usual brand cigarette (n = 30)	THP 1.0(RT) (n = 31)	THP 1.1(RT) (n = 30)	NRT (inhalator) (n = 30)
**C**_**max**_** (ng/mL)**				
Geometric mean	22.7	8.6	10.5	2.3
Geometric mean CV%	94.95	89.65	99.69	61.40
**AUC**_**0–240**_ (ng*min/mL)				
Geometric mean	1316.9	519.2	670.8	333.3
Geometric mean CV%	63.44	69.08	77.12	64.74
**T**_**max**_**, (min)**				
Median	6.03	4.05	4.07	15.03
Range: Min – Max	3.0–9.1	1.1–45.0	1.2–15.4	1.0–91.7

Seven subjects were excluded from the PK analysis population due to major protocol deviations (washout problem), therefore n = 30 for usual brand cigarette, n = 31 for glo THP1.0(RT), n = 30 for glo THP1.1(RT) and n = 30 for Nicorette 15 mg Inhalator.

*CV* coefficient of variation, *Max* maximum, *Min* minimum, *THP* tobacco heating product, *NRT* nicotine replacement therapy.

For both AUC_0-240 min_ and C_max_, THP1.0(RT) and THP1.1(RT) were found to be non-inferior to the NRT, and the cigarette non-inferior to either THP, meaning that the amount and maximum concentration of nicotine in the blood following use of either THP were between levels found for the NRT and the cigarette. Both THPs were superior to the NRT for T_max_, i.e. absorption of nicotine was faster after use of the THPs than after use of the NRT (Table 3).

**Table 3:** Comparison of plasma nicotine pharmacokinetic parameters.

Parameter	Geometric LS mean	Geometric LS mean 90% CI	Ratio^a^	Ratio 90% CI	*P* value	Non-inferiority test^b^
**C**_**max**_** (ng/mL)**						
THP1.0(RT)	8.71	(6.93, 10.95)	3.89	(3.262, –)	< 0.0001	Accepted
NRT	2.24	(1.78, 2.82)				
THP1.0(RT)	8.71	(6.93, 10.95)	0.37	(–, 0.446)	< 0.0001	Accepted
Cigarette	23.27	(18.46, 29.33)				
THP1.1(RT)	10.87	(8.63, 13.70)	4.86	(4.064, –)	< 0.0001	Accepted
NRT	2.24	(1.78, 2.82)				
THP1.1(RT)	10.87	(8.63, 13.70)	0.47	(–, 0.558)	< 0.0001	Accepted
Cigarette	23.27	(18.46, 29.33)				
**AUC**_**0–240**_ (ng*min/mL)						
THP1.0(RT)	527.1	(438.75, 633.35)	1.54	(1.383, –)	< 0.0001	Accepted
NRT	341.4	(283.77, 410.64)				
THP1.0(RT)	527.1	(438.75, 633.35)	0.38	(–, 0.428)	< 0.0001	Accepted
Cigarette	1374.2	(1142.37, 1653.11)				
THP1.1(RT)	694.7	(577.57, 835.58)	2.04	(1.821, –)	< 0.0001	Accepted
NRT	341.4	(283.77, 410.64)				
THP1.1(RT)	694.7	(577.57, 835.58)	0.51	(–, 0.565)	< 0.0001	Accepted
Cigarette	1374.2	(1142.37, 1653.11)				
**T**_**max**_**, (min)**						
THP1.0(RT)			-14.07	(-23.48, -7.55)	< 0.0001	Accepted
NRT						
THP1.1(RT)			-17.30	(-22.99, -7.92)	< 0.0001	Accepted
NRT						

### Subjective responses to product use

To assess different aspects of abuse liability, four questionnaires assessing product liking (PLQ), overall intent to use again (OIUA), urge to smoke (UTS) and urge for product (UFP) were completed before, during, and at various timepoints after study product use. Regarding product liking the mean PLQ (AUC_3-240 min_) score was higher for the cigarette than for the other study products, higher for the two THPs than for the NRT, and was slightly higher for THP1.1(RT) than for THP1.0(RT) (Table 4). In the test for non-inferiority, the null hypothesis was rejected and therefore it can be concluded that neither THP was found to be inferior to the NRT in terms of product liking (Table 5).

**Table 4:** Mean (SD) questionnaire scores for the four investigational products.

Questionnaire	Cigarette (n = 27)	THP1.0(RT) (n = 31)	THP1.1(RT) (n = 29)	NRT (n = 30)
PLQ AUC_3–240_	2107.2 (402.54)	719.5 (732.79)	820.2 (723.78)	356.1 (473.83)
OIUA	9.1 (1.37)	2.5 (2.67)	3.1 (2.84)	1.0 (1.77)
UTS AUC_3–240_	1434.4 (483.3)	1667.3 (559.76)	1603.1 (573.62)	1651.0 (571.00)
Mean UFP^a^	–	2.3 (2.28)	2.8 (2.37)	1.5 (1.89)

Eleven subjects were excluded from the PP population due to major protocol deviations (7 × washout problem, 5 × questionnaire timing out of tolerance), therefore n = 27 for usual brand cigarette, n = 31 for glo THP1.0(RT), n = 29 for glo THP1.1(RT) and n = 30 for Nicorette 15 mg Inhalator.

*OIUA* overall intent to use again, *PLQ* product liking questionnaire, *UFP* urge for product, *UTS* urge to smoke, *THP* tobacco heating product, *NRT* nicotine replacement therapy.

^a^Mean of scores recorded at 15 and 120 min.

**Table 5:** Comparison of primary efficacy variables between each THP and the NRT.

Parameter	Geometric LS mean	Geometric LS mean 90% CI	Ratio^a^	Ratio 90% CI	*P* value	Non-inferiority test^ b^	Superiority test^ c^
**PLQ (AUC**_**3–240 min**_**)**							
THP1.0(RT)	94.0	(38.50, 229.30)	2.61	(1.227, –)	0.0235	Accepted	
NRT	36.0	(14.60, 88.69)					
THP1.1(RT)	191.5	(76.49, 479.17)	5.32	(2.451, –)	0.0012	Accepted	
NRT	36.0	(14.60, 88.69)					
**OUIA (actual values)**							
THP1.0(RT)	2.48	(1.98, 3.10)	1.61	(1.395, –)	< 0.0001	Accepted	
NRT	1.54	(1.23, 1.93)					
THP1.1(RT)	2.98	(2.37, 3.74)	1.94	(1.670, –)	< 0.0001	Accepted	
NRT	1.54	(1.23, 1.93)					
**UTS (AUC**_**3-240 min**_**)**							
THP1.0(RT)	1570.3	(1381.55, 1784.79)	1.01	(–, 1.084)	0.5816		Rejected
NRT	1552.9	(1365.51, 1765.96)					
THP1.1(RT)	1492.8	(1311.34, 1699.44)	0.96	(–, 1.033)	0.2397		Rejected
NRT	1552.9	(1365.51, 1765.96)					
**Mean UFP**^** d**^							
THP1.0(RT)	2.54	(2.04, 3.17)	1.35	(1.152, –)	0.0087		Accepted
NRT	1.88	(1.51, 2.36)					
THP1.1(RT)	3.09	(2.46, 3.87)	1.64	(1.392, –)	0.0001		Accepted
NRT	1.88	(1.51, 2.36)					

Parameters were log transformed and 1 was added to avoid a logarithm of 0. The transformed parameters were analysed by an ANOVA model, including treatment, sequence and period as fixed effects, and subject-within-sequence as a random effect.

*CI* confidence interval, *LS* least squares, *THP* tobacco heating product, *NRT* nicotine replacement therapy.

^a^Calculated by transforming the difference between the natural log LS means back to the original scale.

^b^PLQ and OIUA were confirmed as non-inferior if the lower bound of the one-sided 90% Cl of the ratio was more than 0.80.

^c^UTS was confirmed as superior if the upper bound of the one-sided 90% Cl of the ratio was less than 1.0. UFP was confirmed as superior if the lower bound of the one-sided 90% Cl of the ratio was more than 1.0.

^d^Mean of the scores recorded at 15 and 120 min.

Regarding participant’s intent to use the product again, the OIUA score was higher for the cigarette than for all other study products, higher for the two THPs than for the NRT, and was slightly higher for THP1.1(RT) than for THP1.0(RT) (Table 4). The null hypothesis was rejected in the non-inferiority test, meaning that neither THP was inferior to the NRT in terms of intent to use the product again (Table 5).

The UTS questionnaire was used to determine whether 5-min use of the study products satisfied the participants urge to smoke. The mean UTS (AUC_3-240 min_) score was lower for the cigarette than for any of the other three study products (Table 4). The criteria for superiority of the THP variants over the NRT in terms of urge to smoke were not satisfied (Table 5). The participants’ urge to use the study products, assessed by the UFP score, was higher for the THP variants than for the NRT (Table 4). In the test for superiority, the null hypothesis was rejected and thus it can be concluded that both THP1.0(RT) and THP1.1(RT) were found to be superior to the NRT in terms of urge to use the product again (Table 5).

### Secondary endpoints

Regarding user behaviour, the number of puffs taken during 5-min product use sessions was similar across the study products. The mean number of puffs during the 5-min use session was 12 ± 5.7 (median 12) for THP1.0(RT), 12 ± 5.2 (12) for THP1.1(RT), 13 ± 5.8 (12) for the NRT, and 14 ± 4.6 (13) for the cigarette.

In terms of product evaluation, the mean scores for single items of the PES indicated better satisfaction both pre-use, and 15 and 240 min post-use, for the cigarette than for the three other study products, and an overall higher level of satisfaction for using both THP1.0(RT) and THP1.1(RT) than for the NRT. Based on the PES scores obtained before each product use session, use of the usual brand cigarette was considered by participants to (1) be more effective in relieving withdrawal symptoms and urge to smoke, (2) contain enough nicotine, (3) cause bothersome side effects, and (4) reduce craving for a cigarette after using the product, as compared with the other study products. Conversely, use of the participant’s usual brand cigarette was perceived to have a higher risk of causing dizziness or dependency. In addition, use of the inhalator NRT was perceived as having a higher risk of causing dizziness and nausea, or containing more nicotine, as compared with either THP1.0(RT) or THP1.1(RT) (Supplementary Table 2).

The most relevant changes reported between pre-use and 240 min after use were (1) a perceived reduction in irritability and urge to smoke for use of the cigarette; (2) a perceived increase in risk of causing dizziness for THP1.1(RT) and the cigarette; and (3) a perceived decrease in risk of causing dizziness for the NRT. The perception of craving for a cigarette decreased between pre-use and 240 min post-use for both THP1.0(RT) and THP1.1(RT).

### Safety assessment

A total of 8 treatment-emergent adverse events (TEAEs) were reported in 6 subjects (18.8%) overall (Supplementary Table 3). Two TEAEs were reported in 2 subjects (6.3%) during the use of glo THP1.0(RT), 2 TEAEs were reported in 2 subjects (6.3%) during the use of glo THP1.1(RT), 1 TEAE was reported in 1 subject (3.1%) during the use of the NRT, and 3 TEAEs were reported in 3 subjects (9.4%) during the use of their usual brand cigarette. One TEAE occurring in 1 subject (3.1%) during the use of their usual brand cigarette was considered as treatment-related (cough). There were no serious adverse events, TEAEs of severe intensity or TEAEs leading to discontinuation of the study products. There were no clinically important changes in systolic blood pressure, diastolic blood pressure, pulse rate, body temperature or respiratory rate from pre-dose to any post-dose time point with any of the study products.

## Discussion

The last decade has seen an increasing number of non-combustible tobacco and nicotine-containing products introduced to the consumer market. These include electronic cigarettes/vaping devices, Tobacco Heating Products (THPs), and oral products which deliver nicotine absent of tobacco. Furthermore, although they are not marketed to compete directly with consumer products, medicinal nicotine replacement therapies (NRT) such as nicotine gums, lozenges, inhalers and transdermal patches are also accessible as an aid to smoking cessation and freely available over-the-counter in many countries. In addition to capitalising on new technologies, the expansion in the number of new consumer nicotine products has been driven by the recognition that harm reduction may be a viable strategy for those who cannot or will not quit smoking combusted tobacco^19^. One such product type, THPs, have been shown to be, or evidence is accumulating which suggest that their use may be, less harmful than cigarette smoking, as estimated by reductions in exposure to known toxicants in cigarette smoke or favourable changes in biomarkers for cancer, cardiovascular disease and chronic obstructive pulmonary disorders^26–30^.

What is less well understood is whether there are differences regarding the abuse liability, and consequently the development of nicotine dependence, associated with the use of THPs, or regarding the abuse liability of THPs relative to combustible cigarettes. Furthermore, the possibility that there will be an indirect effect of alternative nicotine products in elevating nicotine use among non-smokers has been raised in the public health community^50^. In consequence, determination of abuse liability is now a regulatory requirement to obtain marketing authorisation for tobacco and nicotine-containing products in some countries, with the expectation that this will give an indication as to the proportion of individuals within a population who will become dependent on a new nicotine product. It is also of importance given the potential impact of alternative nicotine products on overall population health for manufacturers to understand the abuse liability of any new product.

The measurement of abuse liability in human subjects is complex. For tobacco and nicotine products, a consensual view is that assessment of abuse liability requires data examining both nicotine PK and subjective effects^31,34–36^. While the specifics of the types of subjective effects measures to assess are less well defined, recent studies on a variety of tobacco and nicotine products have examined measures of product liking, urge to smoke or to use the product again, and intent to use again, as well as assessing indices related to product use such as enjoyment, satisfaction, relief, and other negative and positive effects^13,14,39,40,50^, in order to generate comparative abuse liability assessments. Integrating these many different inputs into an overall abuse liability assessment commonly also involves comparing abuse liability of control products with high (cigarettes) and low (NRT inhaler) abuse liability^34,45,47^ and, taking all factors into account, providing a subjective assessment of relative abuse liability.

Overall, utilising such an integrated approach combining both nicotine PK and subjective effects, data obtained in this study demonstrate an abuse liability of the THP variants which is lower than that of participants’ usual brand cigarette yet higher than that of NRT inhaler. In terms of nicotine PK, the cigarette had approximately twofold higher C_max_ than the two THPs; however, mean T_max_ was similar for the THP compared with the cigarette, suggesting that nicotine absorption was similar between the two products. Notably, the NRT inhaler, a licensed medical product designed to relieve and/or prevent nicotine cravings and withdrawal symptoms associated with tobacco dependence and to promote smoking cessation, had an approximately fourfold lower C_max_ and fourfold longer T_max_ compared with the THPs. This indicates that because THPs more closely mimic the nicotine delivery of cigarettes than do NRT inhaler’s, smokers may find THP’s more acceptable as alternatives to cigarettes. These findings were supported by the subjective response questionnaires, which showed that the THPs were non-inferior to the NRT inhaler in terms of PLQ and OIUA scores, and superior to the NRT inhaler in terms of UFP score.

In terms of tobacco harm reduction, the abuse liability profile of THPs demonstrated here supports their role reducing in smoking-related harms. A product with low abuse liability likely will not be adopted or used extensively and may not encourage existing smokers to switch from using high-harm cigarettes to lower-harm THPs, and some degree of abuse liability of nicotine products has been proposed to support overall population tobacco harm reduction^44,45^. The potential trade-off of this abuse liability may be the potential for initiation and sustained use among nicotine non-users, or relapse into tobacco use by former smokers^44,45,50^. However, studies have shown that, for THPs, use is extremely uncommon (0.1%) among those with no smoking history while use among former smokers is also very uncommon (1%)^52^.

Until now only one study has examined the abuse liability^42^, and only one study has reported the nicotine PK^41^, of THPs. Thus, a major strength of this study is our rigorous assessment of the abuse liability of a THP in comparison to cigarettes and NRT inhaler, which was conducted in accordance with consensus standards for tobacco product abuse liability assessments. A further strength was the use of overnight confinement of participants within the clinic to ensure compliance with PK assessment procedures and abstention from tobacco product use. Limitations include assessment only of a single type of THP, and as such our findings may not be generalisable to other THPs currently marketed in many countries. Furthermore, only a single THP flavour (tobacco) was assessed and although flavour has been shown not to impact abuse liability of e-cigarettes^13,14,39,40^, we are unable to discern whether non-tobacco flavoured THPs have a different level of abuse liability. Lastly, the study is also limited by a single session which does not necessarily mimic real word product use where THP can have multiple sessions.

In conclusion, we have described findings from a study which rigorously assessed the abuse liability of a THP, demonstrating that the glo THP has a lower abuse liability than cigarettes but a higher abuse liability than NRT inhaler. Since THPs have lower emissions and reduced in vitro biological activity compared to cigarettes, and since switching to using THPs reduces exposure to harmful toxicants, the presence of some degree of abuse liability of the THP supports tobacco harm reduction such that it may provide an appealing and accepting alternative to cigarette smoking in adult smokers and be supportive of their switching away from harmful cigarette smoking.

## Supplementary Information

Supplementary Tables.

## Data Availability

The datasets generated during and/or analysed during the current study are available from the corresponding author on reasonable request.
