# Author Correction: An abuse liability assessment of the glo tobacco heating product in comparison to combustible cigarettes and nicotine replacement therapy

**DOI:** 10.1038/s41598-023-37432-2

**Published:** 2023-06-27

**Authors:** George Hardie, Nathan Gale, Michael McEwan, Stefano Milleri Oscar, Luigi Ziviani, Christopher J. Proctor, James Murphy

**Affiliations:** 1 British American Tobacco (Investments) Limited, Research and Development, Regents Park Road, Southampton, SO15 8TL UK; 2 Centro Ricerche Cliniche di Verona, Policlinico G. B. Rossi, P.Le L. A. Scuro 10, 37134 Verona, Italy; 3 DoctorProctorScience Limited, 157 Cavendish Meads, Sunninghill, Ascot, SL5 9TG UK; 4 R. J. Reynolds Tobacco Company, 401 N Main Street, Winston Salem, NC 27101 USA

Correction to: *Scientific Reports* 10.1038/s41598-022-19167-8, published online 29 August 2022

In the original version of this Article, the Supplementary Information file was omitted from the Supplementary Information section.

The Supplementary Information file now accompanies the original Article.

