# Supplementary material for: An abuse liability assessment of the glo tobacco heating product in comparison to combustible cigarettes and nicotine replacement therapy
Source: Sci Rep. 2022 Aug 29;12:14701. doi: 10.1038/s41598-022-19167-8 (PMC9424205; doi:10.1038/s41598-022-19167-8)
Supplement: Supplementary file 1 — Supplementary Tables. [file 41598_2022_19167_MOESM1_ESM.pdf]

Supplementary file for "An abuse liability assessment of the glo tobacco heating product in comparison to combustible cigarettes and nicotine replacement therapy"

George Hardie, Nathan Gale, Michael McEwan, Stefano Milleri Oscar, Luigi Ziviani, Christopher J. Proctor and James Murphy

Supplementary Table 1. Sample size rationale and assumptions. *AUC* area under the concentration time curve, *NRT* nicotine replacement therapy, *THP* tobacco heating product, *CV* coefficient of variation, C_{max} maximum plasma nicotine concentration, T_{max} time of the maximum plasma nicotine concentration.

Endpoint	Assumptions	Power	Sample Size
AUC_{nic} Log-normal	THP is expected to reach a non-inferior AUC than the NRT where inferiority margin is set at geometric mean ratio of 0.8. At the same time the geometric mean ratio between the THP and usual brand cigarette is expected to not be above 1.25 (α = 0.1). Based on a CV = 0.4 and correlation between pairs observations of 0.2, with observed mean ratio between THP and NRT of 1 and 0.8 between THP and usual brand cigarette.	0.902	32
C_{max} Log-normal	THP is expected to reach a non-inferior C_{max} than the NRT where inferiority margin is set at geometric mean ratio of 0.8. At the same time the geometric mean ratio between the THP and standard cigarette is expected not to be above 1.25 (α = 0.1). Based on group CVs = 0.58 and 0.44 for THP and standard cigarette. Correlation between pairs observations of 0.2, with observed mean ratio between THP and NRT is expected to be approximately 1.5 and 0.8 between THP and standard cigarette.	0.917	14
T_{max} Normal	Mean difference of T_{max} for THP with respect to NRT is expected to be larger than 4 minutes, i.e. at least 4 min earlier for THP than for NRT. Assuming an observed difference of 7 and SD = 5 (α = 0.1).	0.907	31
Overall intent to use again Log-normal	It is assumed that the overall intent to use the THP again is not inferior to NRT. There is no previous data available for this endpoint but assuming geometric mean ratio = 1 and CV = 0.4, with α = 0.1.	0.902	32
Product liking Log-normal	It is assumed that THP product liking is not inferior to NRT. There is no previous data available for this endpoint but assuming geometric mean ratio = 1 and CV = 0.4, with α = 0.1.	0.902	32
Urge to smoke Log-normal	Urge to smoke is assumed to be reduced by the THP compared to NRT. With observed geometric ratio of 0.8.	0.902	32
Urge for product Log-normal	Urge to use the product is assumed to be greater with the THP than with the NRT. With observed geometric ratio of 1.25.	0.902	32

Supplementary Table 2. Summary of Product Evaluation Scale (PES) responses. *THP* tobacco heating product, *N* number of subjects.

PES question	Timepoint	Statistic	THP1.0(RT) (N=32)	THP1.1(RT) (N=32)	Nicorette Inhalator (N=32)	Usual brand cigarette (N=32)
1. Was it satisfying?	Pre-use	Mean (SD)	3.1 (2.22)	3.1 (2.23)	1.9 (1.17)	6.7 (0.87)
		Median	2.5	2.5	1.0	7.0
		Min – Max	1 – 7	1 – 7	1 – 5	3 – 7
		Q1 – Q3	1 – 5	1 – 5	1 – 3	7 – 7
	15 min	Mean (SD)	3.0 (2.14)	3.2 (1.97)	1.9 (1.20)	6.3 (1.30)
		Median	3.0	2.5	1.0	7.0
		Min – Max	1 – 7	1 – 7	1 – 5	2 – 7
		Q1 – Q3	1 – 5	2 – 5	1 – 3	6 – 7
	240 min	Mean (SD)	2.9 (2.08)	3.2 (1.89)	1.8 (1.08)	6.4 (0.91)
		Median	2.0	3.0	1.0	7.0
		Min – Max	1 – 7	1 – 7	1 – 5	3 – 7
		Q1 – Q3	1 – 4	2 – 5	1 – 2	6 – 7
2. Did it taste good?	Pre-use	Mean (SD)	2.8 (2.30)	3.0 (2.21)	1.4 (0.80)	6.5 (0.88)
		Median	1.0	2.0	1.0	7.0
		Min – Max	1 – 7	1 – 7	1 – 4	4 – 7
		Q1 – Q3	1 – 5	1 – 5	1 – 2	6 – 7
	15 min	Mean (SD)	2.8 (2.17)	3.0 (2.06)	1.7 (1.07)	5.8 (1.28)
		Median	1.5	2.0	1.0	6.0
		Min – Max	1 – 7	1 – 7	1 – 4	3 – 7
		Q1 – Q3	1 – 4	1 – 5	1 – 2	5 – 7
	240 min	Mean (SD)	2.6 (2.12)	2.8 (1.97)	1.7 (1.14)	6.3 (0.95)
		Median	1.5	2.0	1.0	7.0
		Min – Max	1 – 7	1 – 7	1 – 5	4 – 7
		Q1 – Q3	1 – 4	1 – 5	1 – 2	6 – 7

PES question	Timepoint	Statistic	THP1.0(RT) (N=32)	THP1.1(RT) (N=32)	Nicorette Inhalator (N=32)	Usual brand cigarette (N=32)
3. Did you enjoy the sensations in your mouth?	Pre-use	Mean (SD)	2.8 (2.17)	3.0 (2.06)	1.4 (0.80)	6.3 (1.00)
		Median	2.0	2.5	1.0	7.0
		Min – Max	1 – 7	1 – 7	1 – 4	4 – 7
		Q1 – Q3	1 – 5	1 – 5	1 – 2	6 – 7
	15 min	Mean (SD)	2.7 (2.04)	2.8 (1.97)	1.6 (0.98)	6.0 (1.31)
		Median	2.0	2.0	1.0	7.0
		Min – Max	1 – 7	1 – 7	1 – 4	3 – 7
		Q1 – Q3	1 – 4	1 – 5	1 – 2	5 – 7
	240 min	Mean (SD)	2.6 (2.06)	2.8 (1.70)	1.7 (1.04)	6.1 (1.13)
		Median	2.0	2.0	1.0	7.0
		Min – Max	1 – 7	1 – 6	1 – 4	3 – 7
		Q1 – Q3	1 – 3	1 – 4	1 – 2	5 – 7
4. Did it calm you down?	Pre-use	Mean (SD)	2.4 (1.74)	2.6 (1.88)	1.9 (1.39)	5.8 (1.75)
		Median	1.5	2.0	1.0	7.0
		Min – Max	1 – 7	1 – 7	1 – 7	1 – 7
		Q1 – Q3	1 – 4	1 – 5	1 – 3	5 – 7
	15 min	Mean (SD)	2.5 (1.87)	2.3 (1.57)	1.8 (1.35)	5.3 (1.87)
		Median	2.0	1.5	1.0	6.0
		Min – Max	1 – 7	1 – 6	1 – 6	1 – 7
		Q1 – Q3	1 – 3	1 – 4	1 – 3	4 – 7
	240 min	Mean (SD)	2.0 (1.59)	2.3 (1.55)	1.6 (0.95)	5.3 (1.89)
		Median	1.0	2.0	1.0	5.5
		Min – Max	1 – 7	1 – 7	1 – 5	1 – 7
		Q1 – Q3	1 – 3	1 – 3	1 – 2	4 – 7

PES question	Timepoint	Statistic	THP1.0(RT) (N=32)	THP1.1(RT) (N=32)	Nicorette Inhalator (N=32)	Usual brand cigarette (N=32)
5. Did it make you feel more awake?	Pre-use	Mean (SD)	1.4 (0.95)	1.7 (1.17)	1.3 (0.67)	4.0 (2.33)
		Median	1.0	1.0	1.0	4.0
		Min – Max	1 – 5	1 – 5	1 – 4	1 – 7
		Q1 – Q3	1 – 1	1 – 2	1 – 1	1 – 7
	15 min	Mean (SD)	1.5 (0.98)	1.6 (1.13)	1.6 (1.34)	3.2 (2.27)
		Median	1.0	1.0	1.0	2.0
		Min – Max	1 – 5	1 – 5	1 – 6	1 – 7
		Q1 – Q3	1 – 2	1 – 2	1 – 2	1 – 5
	240 min	Mean (SD)	1.6 (1.11)	1.6 (1.18)	1.5 (1.02)	3.5 (2.05)
		Median	1.0	1.0	1.0	3.0
		Min – Max	1 – 5	1 – 6	1 – 6	1 – 7
		Q1 – Q3	1 – 2	1 – 2	1 – 2	2 – 5
6. Did it make you feel less irritable?	Pre-use	Mean (SD)	1.8 (1.52)	1.9 (1.27)	1.5 (0.92)	4.9 (1.81)
		Median	1.0	1.0	1.0	5.0
		Min – Max	1 – 6	1 – 5	1 – 5	1 – 7
		Q1 – Q3	1 – 2	1 – 3	1 – 2	4 – 7
	15 min	Mean (SD)	1.7 (1.35)	2.0 (1.56)	1.6 (1.13)	4.2 (2.14)
		Median	1.0	1.0	1.0	4.0
		Min – Max	1 – 6	1 – 6	1 – 5	1 – 7
		Q1 – Q3	1 – 2	1 – 3	1 – 2	2 – 6
	240 min	Mean (SD)	1.7 (1.26)	1.7 (1.17)	1.5 (0.92)	4.1 (2.30)
		Median	1.0	1.0	1.0	4.0
		Min – Max	1 – 6	1 – 5	1 – 5	1 – 7
		Q1 – Q3	1 – 2	1 – 2	1 – 2	2 – 7

PES question	Timepoint	Statistic	THP1.0(RT) (N=32)	THP1.1(RT) (N=32)	Nicorette Inhalator (N=32)	Usual brand cigarette (N=32)
7. Did it help you concentrate?	Pre-use	Mean (SD)	1.6 (1.21)	1.7 (1.15)	1.2 (0.66)	3.7 (2.32)
		Median	1.0	1.0	1.0	4.0
		Min – Max	1 – 6	1 – 5	1 – 4	1 – 7
		Q1 – Q3	1 – 2	1 – 2	1 – 1	1 – 6
	15 min	Mean (SD)	1.7 (1.38)	1.8 (1.26)	1.4 (0.80)	3.5 (2.09)
		Median	1.0	1.0	1.0	3.5
		Min – Max	1 – 6	1 – 5	1 – 4	1 – 7
		Q1 – Q3	1 – 2	1 – 2	1 – 2	2 – 5
	240 min	Mean (SD)	1.6 (1.31)	1.8 (1.49)	1.3 (0.78)	3.5 (2.24)
		Median	1.0	1.0	1.0	3.0
		Min – Max	1 – 6	1 – 7	1 – 5	1 – 7
		Q1 – Q3	1 – 2	1 – 2	1 – 1	2 – 5
8. Did it reduce your hunger for food?	Pre-use	Mean (SD)	1.8 (1.36)	2.1 (1.57)	1.3 (0.80)	4.4 (2.30)
		Median	1.0	1.0	1.0	4.0
		Min – Max	1 – 6	1 – 7	1 – 5	1 – 7
		Q1 – Q3	1 – 3	1 – 3	1 – 1	3 – 7
	15 min	Mean (SD)	1.7 (1.12)	2.1 (1.40)	1.7 (1.31)	4.0 (2.19)
		Median	1.0	2.0	1.0	4.0
		Min – Max	1 – 4	1 – 7	1 – 6	1 – 7
		Q1 – Q3	1 – 3	1 – 3	1 – 2	2 – 6
	240 min	Mean (SD)	1.8 (1.14)	2.0 (1.40)	1.5 (0.72)	3.8 (2.07)
		Median	1.0	2.0	1.0	4.0
		Min – Max	1 – 5	1 – 7	1 – 4	1 – 7
		Q1 – Q3	1 – 3	1 – 3	1 – 2	2 – 6

PES question	Timepoint	Statistic	THP1.0(RT) (N=32)	THP1.1(RT) (N=32)	Nicorette Inhalator (N=32)	Usual brand cigarette (N=32)
9. Did it make you dizzy?	Pre-use	Mean (SD)	1.3 (0.93)	1.3 (0.73)	1.8 (1.60)	2.0 (1.62)
		Median	1.0	1.0	1.0	1.0
		Min – Max	1 – 5	1 – 4	1 – 7	1 – 6
		Q1 – Q3	1 – 1	1 – 1	1 – 2	1 – 3
	15 min	Mean (SD)	1.5 (1.16)	1.9 (1.66)	1.4 (0.95)	3.0 (2.43)
		Median	1.0	1.0	1.0	2.0
		Min – Max	1 – 6	1 – 7	1 – 5	1 – 7
		Q1 – Q3	1 – 1	1 – 2	1 – 2	1 – 5
	240 min	Mean (SD)	1.3 (0.62)	1.6 (1.32)	1.4 (0.91)	2.7 (2.35)
		Median	1.0	1.0	1.0	1.0
		Min – Max	1 – 3	1 – 7	1 – 5	1 – 7
		Q1 – Q3	1 – 1	1 – 2	1 – 2	1 – 5
10. Did it make you nauseous?	Pre-use	Mean (SD)	1.2 (0.54)	1.3 (1.18)	1.4 (1.16)	1.1 (0.30)
		Median	1.0	1.0	1.0	1.0
		Min – Max	1 – 3	1 – 7	1 – 6	1 – 2
		Q1 – Q3	1 – 1	1 – 1	1 – 1	1 – 1
	15 min	Mean (SD)	1.2 (0.45)	1.2 (0.49)	1.2 (0.64)	1.8 (1.86)
		Median	1.0	1.0	1.0	1.0
		Min – Max	1 – 3	1 – 3	1 – 4	1 – 7
		Q1 – Q3	1 – 1	1 – 1	1 – 1	1 – 2
	240 min	Mean (SD)	1.1 (0.42)	1.2 (0.75)	1.3 (0.80)	1.4 (1.34)
		Median	1.0	1.0	1.0	1.0
		Min – Max	1 – 3	1 – 5	1 – 4	1 – 7
		Q1 – Q3	1 – 1	1 – 1	1 – 1	1 – 1

PES question	Timepoint	Statistic	THP1.0(RT) (N=32)	THP1.1(RT) (N=32)	Nicorette Inhalator (N=32)	Usual brand cigarette (N=32)
11. Did it immediately relieve your craving for a cigarette?	Pre-use	Mean (SD)	2.7 (1.81)	3.3 (2.18)	2.3 (1.85)	5.2 (2.48)
		Median	2.0	3.0	1.5	7.0
		Min – Max	1 – 7	1 – 7	1 – 7	1 – 7
		Q1 – Q3	1 – 4	1 – 5	1 – 3	4 – 7
	15 min	Mean (SD)	2.7 (1.80)	3.0 (2.00)	2.3 (1.80)	5.5 (2.09)
		Median	2.0	2.0	1.5	7.0
		Min – Max	1 – 7	1 – 7	1 – 7	1 – 7
		Q1 – Q3	1 – 4	1 – 5	1 – 3	5 – 7
	240 min	Mean (SD)	2.5 (1.78)	3.1 (1.83)	2.4 (1.43)	5.3 (2.18)
		Median	2.0	3.0	2.0	6.0
		Min – Max	1 – 7	1 – 7	1 – 7	1 – 7
		Q1 – Q3	1 – 3	2 – 5	1 – 3	4 – 7
12. Did you enjoy it?	Pre-use	Mean (SD)	3.0 (2.28)	3.1 (2.25)	1.7 (1.12)	6.6 (1.16)
		Median	2.0	2.5	1.0	7.0
		Min – Max	1 – 7	1 – 7	1 – 4	1 – 7
		Q1 – Q3	1 – 5	1 – 5	1 – 3	7 – 7
	15 min	Mean (SD)	2.9 (2.12)	3.1 (2.15)	1.8 (1.22)	6.2 (1.20)
		Median	2.0	2.0	1.0	7.0
		Min – Max	1 – 7	1 – 7	1 – 5	2 – 7
		Q1 – Q3	1 – 4	1 – 5	1 – 3	6 – 7
	240 min	Mean (SD)	2.8 (2.05)	3.1 (2.02)	1.6 (0.98)	6.3 (1.06)
		Median	2.0	2.5	1.0	7.0
		Min – Max	1 – 7	1 – 7	1 – 4	3 – 7
		Q1 – Q3	1 – 4	1 – 5	1 – 2	6 – 7

**PES question**	**Timepoint**	**Statistic**	**THP1.0(RT) (N=32)**	**THP1.1(RT) (N=32)**	**Nicorette Inhalator (N=32)**	**Usual brand cigarette (N=32)**
13. Did it relieve withdrawal symptoms?	Pre-use	Mean (SD)	2.5 (1.80)	3.2 (2.11)	2.5 (1.88)	6.3 (1.26)
		Median	2.0	3.0	2.0	7.0
		Min – Max	1 – 7	1 – 7	1 – 7	2 – 7
		Q1 – Q3	1 – 3	1 – 4	1 – 4	6 – 7
	15 min	Mean (SD)	2.7 (1.87)	3.6 (2.11)	2.7 (1.69)	6.2 (1.42)
		Median	2.0	3.0	2.0	7.0
		Min – Max	1 – 7	1 – 7	1 – 7	2 – 7
		Q1 – Q3	1 – 4	2 – 6	1 – 4	6 – 7
	240 min	Mean (SD)	2.8 (1.93)	3.6 (1.87)	2.3 (1.28)	6.3 (1.40)
		Median	2.0	4.0	2.0	7.0
		Min – Max	1 – 7	1 – 7	1 – 5	1 – 7
		Q1 – Q3	1 – 4	2 – 5	1 – 3	6 – 7
14. Did it relieve the urge to smoke?	Pre-use	Mean (SD)	2.6 (1.91)	3.3 (2.18)	2.5 (1.81)	5.6 (2.37)
		Median	2.0	3.0	2.0	7.0
		Min – Max	1 – 7	1 – 7	1 – 7	1 – 7
		Q1 – Q3	1 – 4	1 – 5	1 – 4	6 – 7
	15 min	Mean (SD)	2.8 (1.97)	3.5 (2.11)	2.6 (1.72)	6.5 (1.41)
		Median	2.0	3.0	2.0	7.0
		Min – Max	1 – 7	1 – 7	1 – 7	1 – 7
		Q1 – Q3	1 – 5	2 – 5	1 – 4	7 – 7
	240 min	Mean (SD)	2.7 (1.86)	3.4 (1.86)	2.5 (1.65)	6.0 (1.86)
		Median	2.0	3.0	2.0	7.0
		Min – Max	1 – 7	1 – 7	1 – 7	1 – 7
		Q1 – Q3	1 – 3	2 – 5	1 – 3	6 – 7

PES question	Timepoint	Statistic	THP1.0(RT) (N=32)	THP1.1(RT) (N=32)	Nicorette Inhalator (N=32)	Usual brand cigarette (N=32)
15. Was it enough nicotine?	Pre-use	Mean (SD)	3.2 (2.09)	4.3 (2.35)	4.7 (2.38)	6.5 (1.19)
		Median	3.0	4.5	4.5	7.0
		Min – Max	1 – 7	1 – 7	1 – 7	3 – 7
		Q1 – Q3	1 – 5	2 – 7	3 – 7	7 – 7
	15 min	Mean (SD)	3.6 (2.29)	4.7 (2.07)	4.5 (2.08)	6.7 (0.69)
		Median	3.5	5.0	4.0	7.0
		Min – Max	1 – 7	1 – 7	1 – 7	4 – 7
		Q1 – Q3	1 – 5	3 – 7	3 – 7	7 – 7
	240 min	Mean (SD)	3.4 (2.27)	4.5 (2.31)	4.4 (2.23)	6.6 (0.95)
		Median	3.0	4.0	4.0	7.0
		Min – Max	1 – 7	1 – 7	1 – 7	3 – 7
		Q1 – Q3	1 – 6	3 – 7	3 – 7	7 – 7
16. Was it too much nicotine?	Pre-use	Mean (SD)	1.4 (0.98)	2.4 (1.98)	3.8 (2.57)	1.8 (1.61)
		Median	1.0	1.0	3.0	1.0
		Min – Max	1 – 5	1 – 7	1 – 7	1 – 6
		Q1 – Q3	1 – 1	1 – 4	1 – 7	1 – 1
	15 min	Mean (SD)	1.4 (0.91)	2.7 (1.69)	3.4 (2.43)	1.8 (1.34)
		Median	1.0	2.0	2.5	1.0
		Min – Max	1 – 4	1 – 6	1 – 7	1 – 5
		Q1 – Q3	1 – 1	1 – 4	1 – 6	1 – 2
	240 min	Mean (SD)	1.6 (1.01)	2.7 (1.77)	3.5 (2.51)	1.7 (1.43)
		Median	1.0	2.0	3.0	1.0
		Min – Max	1 – 4	1 – 6	1 – 7	1 – 6
		Q1 – Q3	1 – 2	1 – 4	1 – 7	1 – 1

PES question	Timepoint	Statistic	THP1.0(RT) (N=32)	THP1.1(RT) (N=32)	Nicorette Inhalator (N=32)	Usual brand cigarette (N=32)
17. Was it easy to use?	Pre-use	Mean (SD)	6.5 (1.16)	6.4 (1.39)	6.7 (0.73)	7.0 (0.18)
		Median	7.0	7.0	7.0	7.0
		Min – Max	2 – 7	2 – 7	4 – 7	6 – 7
		Q1 – Q3	7 – 7	7 – 7	7 – 7	7 – 7
	15 min	Mean (SD)	6.2 (1.48)	6.0 (1.96)	6.3 (1.61)	6.8 (0.78)
		Median	7.0	7.0	7.0	7.0
		Min – Max	2 – 7	1 – 7	1 – 7	3 – 7
		Q1 – Q3	6 – 7	6 – 7	7 – 7	7 – 7
	240 min	Mean (SD)	6.2 (1.60)	6.2 (1.43)	6.3 (1.65)	6.8 (0.75)
		Median	7.0	7.0	7.0	7.0
		Min – Max	1 – 7	2 – 7	1 – 7	4 – 7
		Q1 – Q3	7 – 7	6 – 7	7 – 7	7 – 7
18. Were there bothersome side effects?	Pre-use	Mean (SD)	1.5 (1.50)	1.5 (1.52)	1.4 (1.19)	1.1 (0.39)
		Median	1.0	1.0	1.0	1.0
		Min – Max	1 – 7	1 – 7	1 – 5	1 – 3
		Q1 – Q3	1 – 1	1 – 1	1 – 1	1 – 1
	15 min	Mean (SD)	1.4 (1.27)	1.4 (1.16)	1.5 (0.92)	1.9 (1.76)
		Median	1.0	1.0	1.0	1.0
		Min – Max	1 – 7	1 – 7	1 – 5	1 – 7
		Q1 – Q3	1 – 1	1 – 1	1 – 2	1 – 2
	240 min	Mean (SD)	1.1 (0.42)	1.4 (1.21)	1.4 (1.21)	1.8 (1.87)
		Median	1.0	1.0	1.0	1.0
		Min – Max	1 – 3	1 – 7	1 – 7	1 – 7
		Q1 – Q3	1 – 1	1 – 1	1 – 1	1 – 1

PES question	Timepoint	Statistic	THP1.0(RT) (N=32)	THP1.1(RT) (N=32)	Nicorette Inhalator (N=32)	Usual brand cigarette (N=32)
19. Were you comfortable using the product in public?	Pre-use	Mean (SD)	6.2 (1.56)	6.0 (1.96)	5.4 (2.29)	6.5 (1.54)
		Median	7.0	7.0	7.0	7.0
		Min – Max	1 – 7	1 – 7	1 – 7	1 – 7
		Q1 – Q3	7 – 7	7 – 7	4 – 7	7 – 7
	15 min	Mean (SD)	6.1 (1.67)	6.2 (1.71)	5.2 (2.47)	6.3 (1.53)
		Median	7.0	7.0	7.0	7.0
		Min – Max	1 – 7	1 – 7	1 – 7	1 – 7
		Q1 – Q3	6 – 7	7 – 7	3 – 7	7 – 7
	240 min	Mean (SD)	5.9 (1.87)	6.0 (1.80)	5.3 (2.40)	6.3 (1.62)
		Median	7.0	7.0	7.0	7.0
		Min – Max	1 – 7	1 – 7	1 – 7	1 – 7
		Q1 – Q3	5 – 7	6 – 7	3 – 7	7 – 7
20. Did you still have a craving for a cigarette after using the product?	Pre-use	Mean (SD)	4.2 (2.54)	4.3 (2.29)	4.2 (2.47)	2.3 (1.94)
		Median	4.0	5.0	4.0	1.0
		Min – Max	1 – 7	1 – 7	1 – 7	1 – 7
		Q1 – Q3	1 – 7	2 – 7	2 – 7	1 – 3
	15 min	Mean (SD)	4.0 (2.09)	3.4 (1.79)	4.2 (2.02)	2.0 (1.60)
		Median	4.0	3.0	4.0	1.0
		Min – Max	1 – 7	1 – 7	1 – 7	1 – 7
		Q1 – Q3	2 – 6	2 – 5	2 – 6	1 – 2
	240 min	Mean (SD)	3.6 (1.95)	3.8 (2.10)	4.4 (2.11)	3.0 (2.24)
		Median	3.0	3.5	4.0	2.0
		Min – Max	1 – 7	1 – 7	1 – 7	1 – 7
		Q1 – Q3	2 – 4	2 – 5	3 – 7	1 – 5

PES question	Timepoint	Statistic	THP1.0(RT) (N=32)	THP1.1(RT) (N=32)	Nicorette Inhalator (N=32)	Usual brand cigarette (N=32)
21. Are you concerned that you would become dependent on this product?	Pre-use	Mean (SD)	1.2 (0.79)	1.1 (0.39)	1.2 (0.72)	3.3 (2.60)
		Median	1.0	1.0	1.0	1.5
		Min – Max	1 – 5	1 – 3	1 – 5	1 – 7
		Q1 – Q3	1 – 1	1 – 1	1 – 1	1 – 7
	15 min	Mean (SD)	1.3 (0.96)	1.1 (0.34)	1.2 (0.75)	3.0 (2.44)
		Median	1.0	1.0	1.0	1.0
		Min – Max	1 – 6	1 – 2	1 – 5	1 – 7
		Q1 – Q3	1 – 1	1 – 1	1 – 1	1 – 5
	240 min	Mean (SD)	1.3 (0.99)	1.4 (1.16)	1.2 (0.75)	2.7 (2.32)
		Median	1.0	1.0	1.0	1.0
		Min – Max	1 – 6	1 – 7	1 – 5	1 – 7
		Q1 – Q3	1 – 1	1 – 1	1 – 1	1 – 5

Supplementary Table 3. Summary of treatment emergent adverse events (Safety Population). *THP* tobacco heating product, *N* number of subjects, *TEAE* treatment emergent adverse event. Data are number of subjects affected (% of subjects affected) [total number of events reported].

	**THP1.0(RT) (N=32)**	**THP1.1(RT) (N=32)**	**Nicorette Inhalator (N=32)**	**Usual brand cigarette (N=32)**	**Overall (N=32)**
TEAEs	2 (6.3%) [2]	2 (6.3%) [2]	1 (3.1%) [1]	3 (9.4%) [3]	6 (18.8%) [8]
Serious TEAEs	—	—	—	—	—
TEAEs Related to Investigational Product	—	—	—	1 (3.1%) [1]	1 (3.1%) [1]
Severe TEAEs	—	—	—	—	—
TEAEs Leading to Study Product Discontinuation	—	—	—	—	—
TEAEs Leading to Death	—	—	—	—	—
